# The H^+^-pyrophosphatase IbVP1 regulates carbon flux to influence the starch metabolism and yield of sweet potato

**DOI:** 10.1038/s41438-020-00454-2

**Published:** 2021-02-01

**Authors:** Weijuan Fan, Yandi Zhang, Yinliang Wu, Wenzhi Zhou, Jun Yang, Ling Yuan, Peng Zhang, Hongxia Wang

**Affiliations:** 1 National Key Laboratory of Plant Molecular Genetics, CAS Center for Excellence in Molecular Plant Sciences, Chinese Academy of Sciences, Shanghai, 200032 China; 2 Shanghai Key Laboratory of Plant Functional Genomics and Resources, Shanghai Chenshan Plant Science Research Center, Chinese Academy of Sciences, Shanghai, 201602 China; 3 University of Chinese Academy of Sciences, Beijing, 100049 China; 4 Department of Plant and Soil Sciences and Kentucky Tobacco Research and Development Center, University of Kentucky, Lexington, KY 40546 USA

**Keywords:** Biotechnology, Molecular engineering

## Abstract

Storage roots of sweet potato are important sink organs for photoassimilates and energy, and carbohydrate metabolism in storage roots affects yield and starch production. Our previous study showed that sweet potato H^+^-pyrophosphatase (IbVP1) plays a vital role in mitigating iron deficiency and positively controls fibrous root growth. However, its roles in regulating starch production in storage roots have not been investigated. In this study, we found that *IbVP1* overexpression in sweet potato improved the photosynthesis ability of and sucrose content in source leaves and increased both the starch content in and total yield of sink tissues. Using ^13^C-labeled sucrose feeding, we determined that *IbVP1* overexpression promotes phloem loading and sucrose long-distance transport and enhances Pi-use efficiency. In sweet potato plants overexpressing *IbVP1*, the expression levels of starch biosynthesis pathway genes, especially *AGPase* and *GBSSI*, were upregulated, leading to changes in the structure, composition, and physicochemical properties of stored starch. Our study shows that the *IbVP1* gene plays an important role in regulating starch metabolism in sweet potato. Application of the *VP1* gene in genetic engineering of sweet potato cultivars may allow the improvement of starch production and yield under stress or nutrient-limited conditions.

## Introduction

Sweet potato (*Ipomoea batatas* (L.) Lam.) is a multifunctional starchy root crop species that is a primary resource for food, feed, and bioindustrial applications. The annual global production of sweet potato is approximately 92 million tons of fresh storage roots (FAOSTAT 2018), which is crucial for food security, malnutrition and poverty alleviation, and bioindustrial development^1,2^. With increasing demand for sweet potato production, storage roots need to be improved, especially in terms of yield and quality, both of which are positively correlated with the acquisition and partitioning of organic carbon^3,4^. In addition to a strong photosynthesis ability of the leaves, an efficient system of sucrose transport from source to sink via stem phloem can ensure sufficient access to carbohydrates^5–7^. Sucrose transporters (SUTs) are responsible for sucrose loading into the complex comprising phloem sieve elements and companion cells. Overexpression of *SUT* genes has been demonstrated to improve plant productivity by effective partitioning of carbohydrates in sinks in rice, pea, and wheat^8–10^. In sweet potato, overexpression of the H^+^-translocating inorganic pyrophosphatase (H^+^-PPase) gene *IbVP1* increases plasma membrane H^+^-ATPase hydrolytic activities, *SUT1* expression, and root growth^11^. Nevertheless, how IbVP1 regulates starch metabolism in sweet potato storage roots has not yet been determined.

H^+^-PPase involves in plant metabolism by hydrolyzing pyrophosphate to form sodium and/or proton gradients that transport ions across the membrane^12,13^. Under abiotic stress conditions, H^+^-PPase gene expression and enzyme activity increase^14–17^. H^+^-PPase affects the transport and accumulation of auxin and sugar during root and shoot growth^18–22^. These phenomena lead to enhanced root and shoot biomass; increased yields of grain, fiber, or fruit; and improved photosynthesis ability, nutrient and water uptake, and CO_2_ fixation (especially under nutrient-deficient conditions, e.g., low NO_3_^−^ and phosphate (Pi))^9,23–25^. Overexpression of H^+^-PPase in *Arabidopsis* results in the upregulated expression of the sucrose proton symporter *SUC1*, suggesting the involvement of H^+^-PPase in the transport of sucrose into the phloem^18,19^.

In a previous study, we showed that overexpressing *IbVP1* in sweet potato improves plant growth and development through altered carbohydrate metabolism in the leaves, transport of polar auxin from source to sink, and rhizosphere acidification to facilitate nutrient acquisition^11^. However, the underlying molecular mechanism for the function of IbVP1 in storage root starch metabolism remains unclear. In this study, we showed that overexpression of *IbVP1* resulted in an increase in starch accumulation in, storage root numbers of and yield of sweet potato. Transgenic sweet potato plants exhibited increased phloem loading and long-distance transportation of sucrose through the acquisition and utilization of activated Pi, thereby increasing the starch content in and yield of the storage roots. Moreover, the starch physicochemical properties, including their composition and structure, were altered due to the increased transcript level of *granule-bound starch synthase I (GBSSI)*. These findings advance our mechanistic understanding of the functions of *IbVP1* in the starch biosynthesis process and provide a new target for adding value to root crops by improving starch content and quality.

## Materials and methods

### Transgenic sweet potato lines

Sweet potato cv. Taizhong 6, one of the edible yellow-fleshed cultivars grown in China, was used as a donor parent for generating transgenic plants. *IbVP1-*overexpressing transgenic lines (IA lines) were produced as described by Fan et al.^11^. In early May 2014–2017, wild-type (WT) plants and transgenic lines were planted in a field at the Wushe Plantation for Transgenic Crops, Shanghai, China (31°13948.0099°N, 121°28912.0099°E).

### Starch isolation from sweet potato storage roots

The storage roots of IA lines and WT plants were harvested after the plants had grown in the field for 5 months. Freshly harvested storage roots of the sweet potato plants were washed, peeled, cut into small slices, suspended in distilled water, and blended with a commercial scale blender. The starchy liquid was passed through a 100 μm sieve, and the starch granules were allowed to sediment. The supernatants were decanted and replaced four times with distilled water. The final supernatants were discarded, and the samples were dried at 40 °C for two days^26^.

### Analysis of sugar and starch contents

Storage roots of WT and IA lines growing in the field for 5 months were harvested, ground into powder, and then heated at 80 °C for approximately 48 h to obtain a constant dry weight (DW). The dried samples (30 mg) were used to determine the sugar and starch contents, as described by Knutson et al.^27^. The starch content was measured by the use of starch kit (Megazyme International Ireland Limited, Wicklow, Ireland). After sugar extraction, the supernatant was centrifuged and transferred into a new glass tube for high-performance liquid chromatography (HPLC) analysis for the identification of the sugar fractions. The different fractions of sugar were identified on the basis of their retention time via known standards. The sugar concentrations were subsequently determined with a standard curve.

### Amylose content measurements

The amylose contents in the starch samples from 5-month-old field-grown WT and IA plants were measured by the colorimetric method by following a previously described protocol^28^. Pure amylose (Type III, Sigma A0512, St. Louis, MO) and amylopectin (Sigma 10118) of potato starch were purchased and used to make known dilutions to establish standard curves.

### Characterization of starch thermal properties

The thermal properties of starch from 5-month-old field-grown WT and IA plants were assessed by the differential scanning calorimeter method (DSC Q2000; TA Instrument, Ltd., Crawley, UK). All the samples were prepared and kept in aluminum sealed pans for 2 h, after which scanning was performed in the temperature range from 30 to 95 °C. An empty, sealed aluminum pan was used as a reference. The measurements were recorded in triplicate for each individual sample.

### Pasting properties of starch

The pasting properties of starch from 5-month-old field-grown WT and transgenic plants were measured using a Rapid Visco-Analyzer (RVA) (RVA-4 series, Newport Scientific, Warriewood, Australia). A starch suspension (5% wt/vol) was produced by mixing in distilled water, and starch viscosity parameters were recorded at various temperatures as described by Zhou et al.^26^.

### Particle size distribution assays

The distribution of granule size of starch from 5-month-old field-grown WT and IA lines were determined by using a Master-sizer (2000 Laser Diffraction Instrument Malvern Instruments, Ltd., Worcestershire, UK) in wet-cell mode as described by Zhou et al.^26^ and Blazek et al.^29^.

### Morphology of starch granules

The granular morphology of the starch from 5-month-old field-grown plants was observed via scanning electron microscopy (SEM). Starch samples were dispersed in distilled water and then spread onto double-sided adhesive tape, air dried, and coated with gold powder. The samples were observed via SEM (JSM6360lV, JEOL, Tokyo, Japan) and imaged. For transmission electron microscopy (TEM), slices of freshly prepared storage roots (1 mm^3^) were glazed with 1,3-diformal propane. Afterward, ultrathin sectioning was performed, and the samples were examined via TEM (Hitachi H7650, Tokyo, Japan).

### Measurements of amylopectin chain length distribution (CLD)

Amylopectin CLD was measured by using the method of Nishi et al.^30^. First, the isolated sweet potato starch was debranched enzymatically for quantification of the chain-length distribution. In brief, 5 mg of sweet potato starch sample was digested with isoamylase enzymes (Sigma; 15284). The debranched starch samples were then used to quantify the amylopectin content using high-performance anion-exchange chromatography with pulsed amperometric detection (HPAEC-PAD).

### Freeze–thaw stability assays

The stability of starch subjected to freeze–thaw cycles from 5-month-old field-grown plants was evaluated by a previously described method^28^. Briefly, 5% starch paste (wt/vol) was liquified by boiling for 20 min. The samples were cooled, after which 0.5 mL of each sample was transformed into new 1.5 mL Eppendorf tubes. The weight of the starch in each sample tube was measured. All the samples were stored at –70 °C overnight and then put into a water bath to thaw for 60 min at 22 °C. Six replications from each starch tube were taken and centrifuged at 8000 х *g* for 10 min at 18 °C. The supernatant was separated, and the remaining paste was weighed. The obtained value (represented as the percent of the initial sample; wt/wt) was considered a measure of syneresis. The tubes were subsequently stored at −70 °C for further freeze–thaw cycles and measurements.

### Phloem loading measurements

The phloem exudate assay was performed on plants grown for 3 months in a greenhouse as described previously^31^, with some modifications. Stems with three unfolded leaves were cut and soaked immediately in a 15 mM EDTA solution. The stems were then recut to pieces 1.5 to 2 cm in length in 15 mM EDTA solution buffer to collect the phloem exudate. After the samples incubated for 12 h in the dark at 25 °C, phloem exudates were concentrated for sucrose measurements via HPLC.

The photoassimilates were labeled with stable ^13^CO_2_ isotopes to investigate the diffusion rate of photoassimilates into the phloem, as described by Gui et al.^32^ and Koubaa et al.^33^. The first unfolded leaf was positioned in a gas-sealed transparent glass chamber filled with ^13^CO_2_. After 12 h of photosynthesis, treated plants were kept in the dark, and leaf samples were collected for sucrose isolation and measurements. Following derivatization, analysis was performed by gas chromatography–mass spectrometry (GC–MS). The proportions of ^13^C isotope mass spectral fragments were determined to measure the amounts of ^13^C-labeled sugars.

### Measurements of leaf area

Leaves from sweet potato plants that had been growing for 3 months (six different pots) were sampled to measure the leaf area. The leaves were imaged, and the leaf area (mm^2^) was measured using WSeen software. At least six replicates for each leaf were recorded for the transgenic lines and WT.

### Photosynthesis measurements

Net photosynthesis was measured on the third leaf from the top of plants grown for three months in the field, using a LI-6400 photosynthesis system (LI-6400 Inc., Lincoln, NE, USA). All measurements were carried out at 9:00 to 10:00 a.m. following the manufacturer’s instructions.

### Sucrose phosphate synthase (SPS) activity assays

For SPS analysis, the first unfolded leaves of 3-month-old plants were frozen in liquid nitrogen, after which total proteins were extracted, desalted using a spin column, and immediately assayed. SPS activity was determined using a sucrose phosphate synthase assay kit (Solarbio BC0600) according to the manufacturer’s specifications.

### Activity assays of storage root enzymes

Vacuole H^+^-PPase activity was determined using the method of Fan et al.^11^.

The enzyme activity of starch biosynthesis-related enzymes (AGPase, SS, GBSS, and SBE) was measured in tuberous roots of the AI and WT plants by following the methods described by Nakamura et al.^34^. One unit of enzyme (SS, GBSS, and AGPase) activity was defined as the production of 1 nmol of ADP per min at 30 °C, while 1 unit of SBE enzyme activity was described as the amount of enzyme needed to increase the spectrophotometric absorbance by one unit at 540 nm in 1 min. The activities of α-amylase and β-amylase in the storage roots of sweet potato plants were also measured. A Ceralpha kit (Megazyme International Ireland, Bray Business Park, Bray, Co., Wicklow, Ireland.) was used following the manufacturer’s protocol, with the appropriate dilutions.

### Expression of starch biosynthesis enzymes

The mRNA levels of starch biosynthesis-related genes in the storage roots of IA and WT plants were measured by quantitative real-time reverse transcription polymerase chain reaction (qRT-PCR). Gene-specific primers were designed (Table S3) to analyze the expression levels using SYBR Green PCR Master Mix (Bio-Rad) in a Bio-Rad CFX96 thermocycler. Amplification was performed under the following conditions: 95 °C for 1 min, followed by 40 cycles of 95 °C for 15 s and 60 °C for 30 s. The relative expression levels of the genes were calculated using the sweet potato *Actin* gene as an internal control.

### Pyrophosphate determination

For the quantification of pyrophosphate (PPi), roots from 5-month-old field-grown plants were collected and immediately frozen in liquid nitrogen to avoid PPi hydrolysis. The frozen roots were homogenized in chilled 80% ethanol, vortexed for 10 min, heated at 80 °C for 30 min, and then centrifuged at 20,000 x *g* for 5 min. The precipitate was subsequently resuspended in chilled 80% ethanol and centrifuged. The supernatants were collected and dried with a centrifugal evaporator. The dried extract was resuspended in water, and water-soluble compounds were extracted. PPi was measured using a pyrophosphate assay kit (Sigma MAK168-1KT) according to the manufacturer’s specifications. All glassware was pretreated overnight with 0.1 M HCl to remove residual Pi.

### Determination of Pi in sweet potato tissue

The Pi content in roots and shoots of 5-month-old field-grown plants was determined by digesting the tissues in H_2_SO_4_–H_2_O_2._ After 2 mL of H_2_SO_4_ was added to the samples followed by thorough shaking (including the blowing of air through a pipette), 1 mL of H_2_O_2_ was added two times via multiple small drops. After the reaction was completed, the sample was placed in an electric heater (nitrogen blowing at 120 °C for 15 min) until the solid disappeared into an essentially transparent solution. The digested solutions were subsequently diluted with ddH_2_O. Aliquots of diluted solutions (0.5 mL) were added to 4 mL of a solution consisting of 0.5% (w/v) ammonium molybdate, 0.6 M H_2_SO_4_, and 2% (w/v) ascorbic acid. The absorbance was read at 660 nm, and the Pi content was measured as milligrams of Pi per gram of fresh weight.

### Statistical analysis

At least three biological replicates were used for each experiment. The data are reported as the means ± SDs, with three replicates for each individual experiment. Significant differences were identified for each individual treatment using Student’s *t* test via SigmaPlot 10.0 (Systat Software, San Jose, CA). The differences among the treatments were statistically significant when *P* was <0.05 or 0.01.

## Results

### *IbVP1-*overexpressing sweet potato lines presented increased yield and higher phosphorus accumulation

**Fig. 1:**
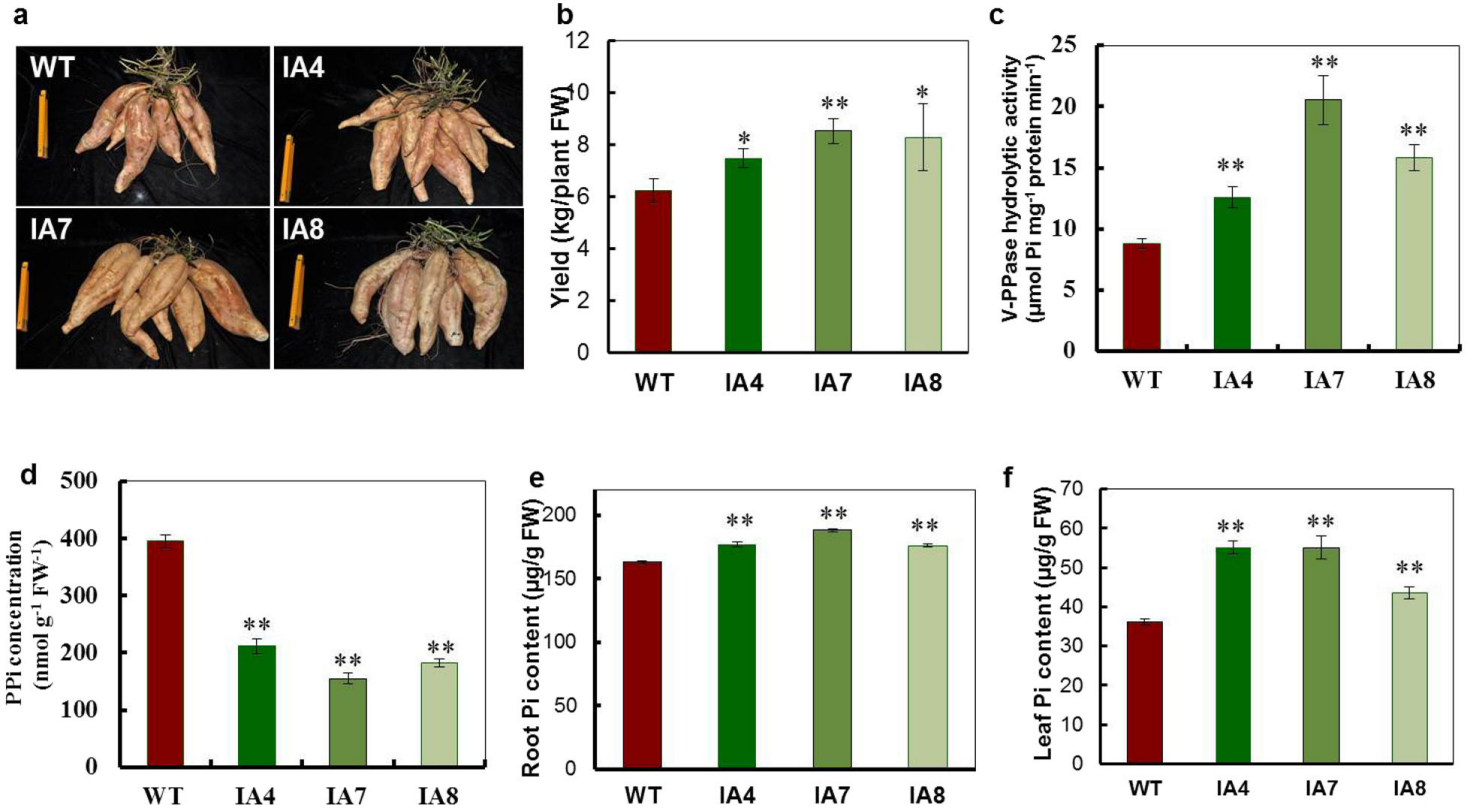
Storage root production of and Pi content in sweet potato. **a** Morphological characteristics of storage roots from harvested wild-type (WT) and *IbVP1*-overexpressing transgenic (IA lines) sweet potato. **b** Yield of storage roots of WT and IA lines. **c** Pyrophosphatase activity and **d** PPi concentrations in the storage roots of WT and IA lines. **e**–**f** Leaf and storage root phosphorus contents in WT and IA lines. Five-month-old storage roots harvested from the field were used for analysis. The asterisks represent significant differences from the WT at *P < 0.05 or **P < 0.01 (Student’s *t* test)

We generated stable *IbVP1* transgenic sweet potato lines and characterized the impacts of *IbVP1* overexpression on shoot and fibrous root growth^11^. However, the storage roots of the *IbVP1* transgenic lines have not been thoroughly studied. To understand the impact of *IbVP1* overexpression on storage root yield, mature storage roots from 5-month-old *IbVP1-*overexpressing sweet potato plants (IA lines) were harvested from the field. Compared with the WT plants, all IA lines exhibited higher yields and increased numbers of storage roots (Fig. 1a, b, Fig. S1a-c). Under field conditions, the yield of the IA lines ranged from 7.47 to 8.53 kg/plant, which is 20–39% higher than the WT yield 6.23 kg/plant (Fig. 1b). IA plants produced a higher number of storage roots 6.3 ± 1.5, 6.3 ± 1.2, and 5.7 ± 1.2 than did the WT plants (3.7 ± 1.2) (Fig. S1c).

IbVP1 hydrolyzes pyrophosphate to Pi. The V-H^+^-PPase hydrolytic activities of IA and WT roots were determined. As shown in Fig. 1c, the H^+^-PPase activity in the IA plants was higher than that in the WT plants. Moreover, the PPi concentrations decreased, but the Pi concentrations increased more in the three IA lines compared to the WT (Fig. 1d–e). Furthermore, the Pi concentrations of the IA leaves were 41% higher than those of the WT leaves (Fig. 1f), indicating that the IA lines have more Pi available in both leaf and root cells. Pi is an indispensable macronutrient essential for plant growth and development^35^. The higher concentrations of Pi in the IA lines occurred simultaneously with improved root growth.

### *IbVP1* overexpression increases carbohydrate export and translocation from source to sink

**Fig. 2:**
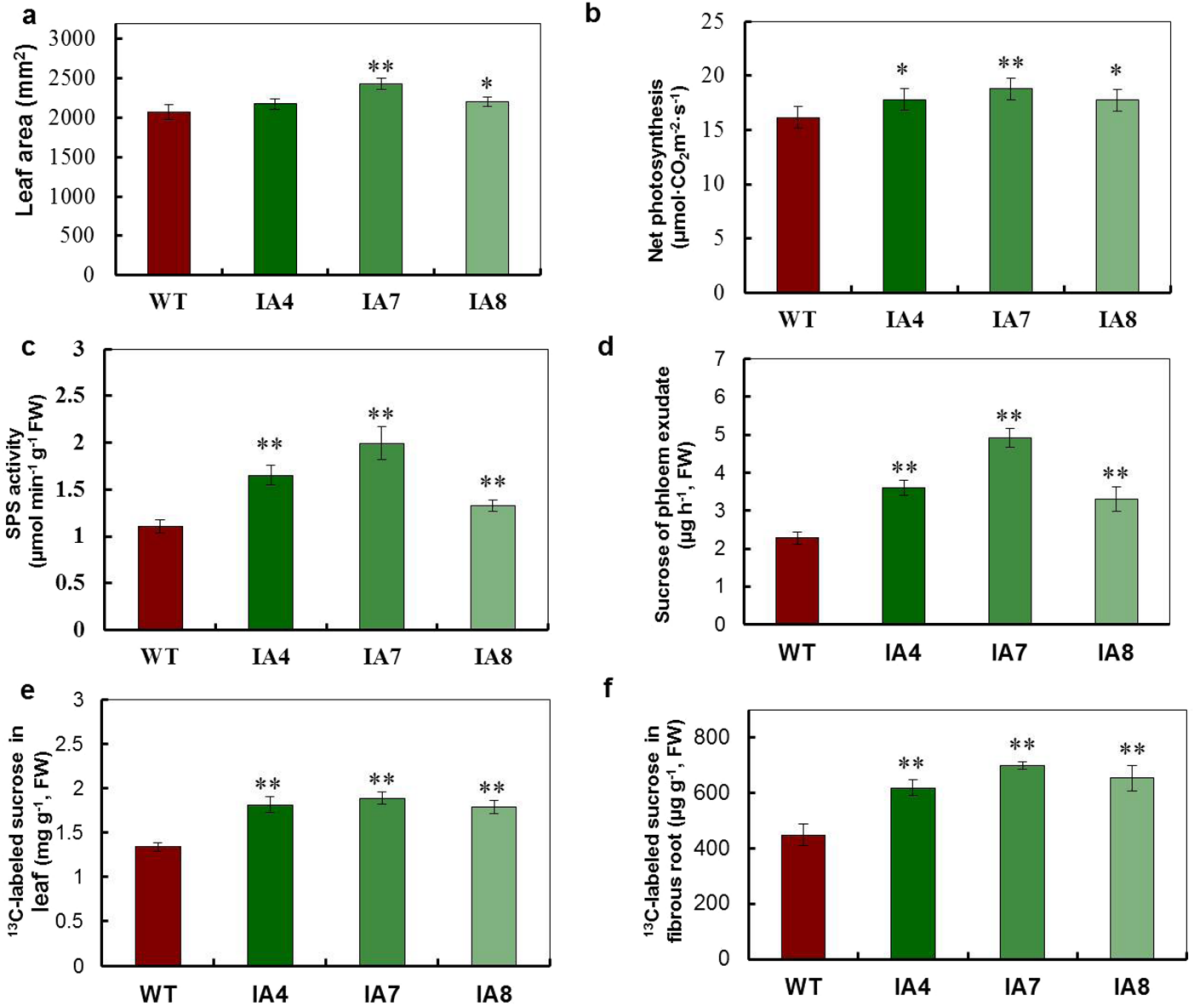
Sucrose translocation in sweet potato. **a** Leaf area, **b** net photosynthesis rate, and **c** SPS activity were measured in wild-type (WT) and IA lines. **d** Sucrose contents in phloem exudates of WT and *IbVP1*-overexpressing transgenic (IA lines) sweet potato. **e**–**f** ^13^C-labeled sucrose in the leaves (**e**) and fibrous roots (**f**) of sweet potato plants after feeding photosynthetic leaves ^13^CO_2_. The asterisks represent significant differences compared to WT at *P < 0.05 or **P < 0.01) (Student’s *t* test)

To assess the source photoassimilates, leaf area and average photosynthesis rates were measured. Compared with the WT, IA7 and IA8 had larger leaf areas (Fig. 2a). The net photosynthesis of the IA leaves was higher than that of WT leaves (Fig. 2b). Sucrose levels significantly increased in the leaves of the IA lines compared with the WT (Fig. S2a). However, the starch contents in the IA leaves were lower than those in the WT leaves (Fig. S2b). Therefore, the ratio of sucrose/starch in the leaves of the IA plants was significantly higher than that of the WT (Fig. S2c). Furthermore, the SPS activities in the IA leaves increased by 1.2- to 1.8-fold, which likely enhanced sugar biosynthesis in the IA leaves compared to WT leaves. To investigate the relationship between photoassimilate translocation and increased yields in the IA plants, we determined the amount of sucrose exported from the leaves. Sucrose levels in phloem exudates collected from the IA lines were significantly higher than those from the WT (Fig. 2c). Additionally, photoassimilate loading from leaves into the phloem was also recorded by using the ^13^CO_2_ stable isotopic labeling method combined with GC–MS. The sucrose contents in the leaves of the IA lines were higher than those in the WT (Fig. 2d). Consistent with these findings, more ^13^C-labeled sucrose was detected in the fibrous roots of IA plants compared with the WT, indicating an increase in the rate of sucrose transport out of leaves of the IA lines (Fig. 2e). Taken together, these results clearly demonstrate that the transgenic IA lines are more efficient at exporting carbohydrates, mainly sucrose, from photosynthetic leaves, promoting the yield of storage roots via upregulation of *IbVP1* expression.

**Fig. 3:**
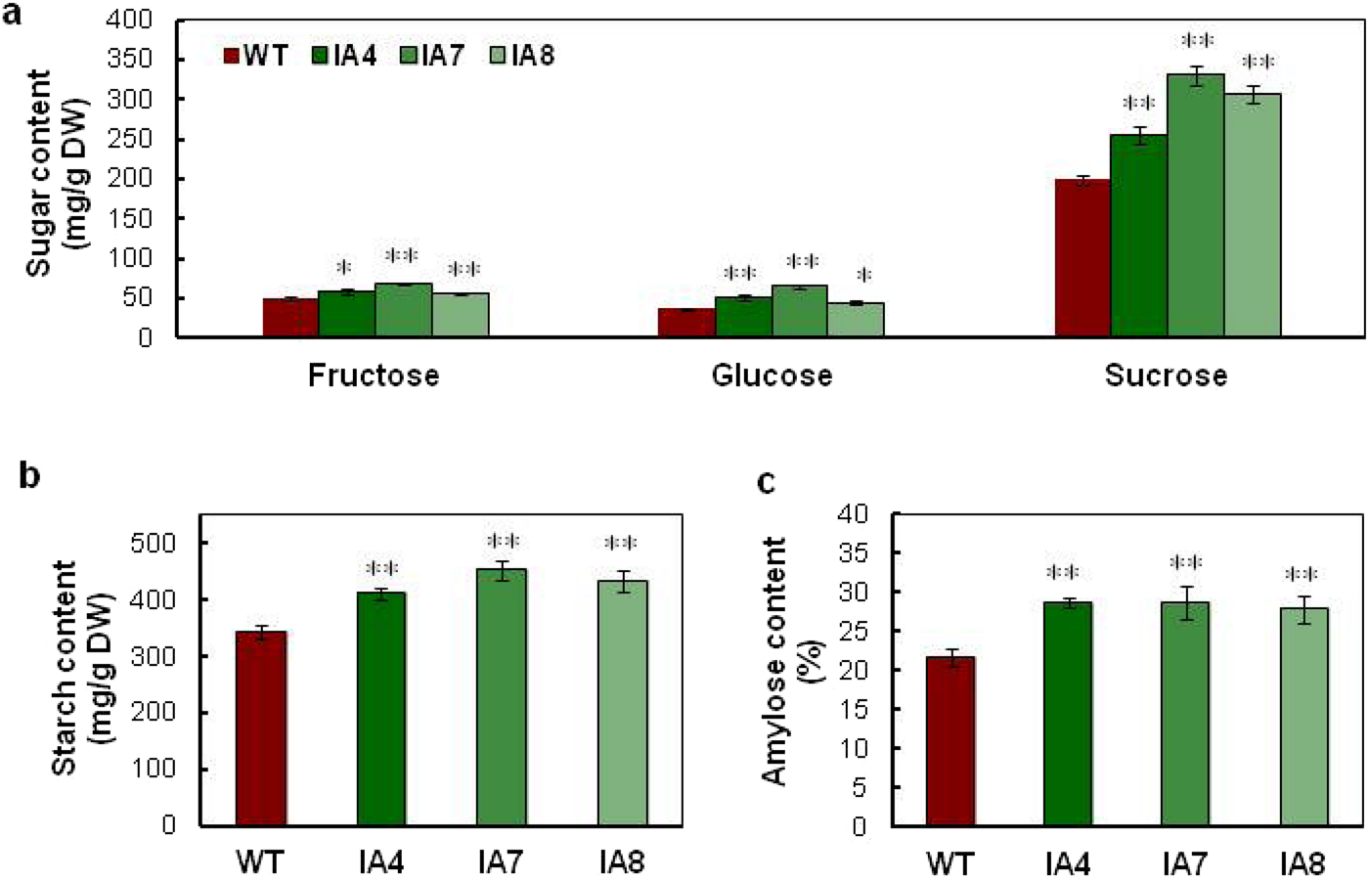
Contents of major carbohydrates in sweet potato storage roots. **a** Fructose, glucose, and sucrose. **b** Starch. **c** Amylose. WT wild type; IA4, IA7, and IA8 are three independent *IbVP1*-overexpressing transgenic lines. Five-month-old storage roots harvested from the field were used for analysis. The asterisks represent significant differences from WT at *P < 0.05 or **P < 0.01 (Student’s *t* test)

Storage root development is strongly associated with starch accumulation^36^. To determine whether starch metabolism is affected by *IbVP1* expression in sweet potato, the levels of major carbohydrates (i.e., fructose, glucose, sucrose, and starch) in the storage roots of IA and WT plants were measured. The sucrose contents in the IA lines were 28–67% higher than those in the WT plants (Fig. 3a). Glucose and fructose were also higher in IA lines than in the controls (Fig. 3a). The total starch and amylose contents in the IA lines were, respectively, 20–32% and 27–32% higher than those in WT, which were significantly different (Fig. 3b–c).

### *IbVP1* overexpression enhances starch biosynthesis ability

**Fig. 4:**
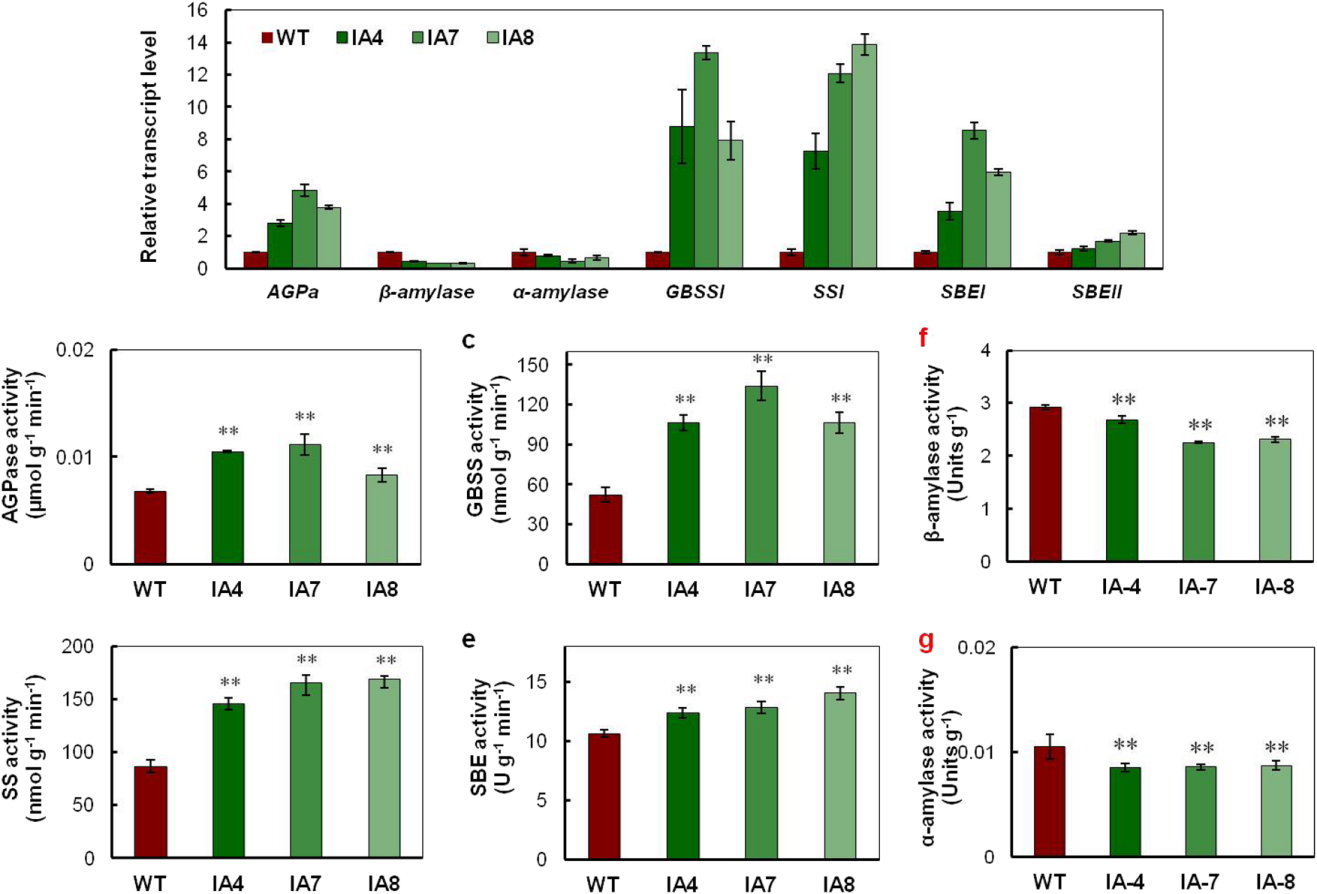
Expression levels and enzyme activities of starch biosynthesis-related genes in sweet potato storage roots. **a** Transcript levels of genes related to starch biosynthesis (*AGPa*, *GBSSI*, *SSI*, *SBEI*, and *SBEII*) and starch degradation (*α-amylase and β-amylase*). **b**–**g** Activities of AGPa, GBSSI, SSI, SBEI, SBEII, α-amylase, and β-amylase. WT wild type; IA4, IA7, and IA8 are three independent *IbVP1*-overexpressing transgenic lines. Five-month-old storage roots harvested from the field were used for analysis. The asterisks represent significant differences from WT at *P < 0.05) or **P < 0.01) (Student’s *t* test)

Since the *IbVP1*-overexpressing sweet potato plants showed an increased carbon flux during starch biosynthesis, we measured the expression of genes involved in starch biosynthesis. We found that the expression levels of the *AGPase*, *GBSSI*, *SSI*, *SBEI*, and *SBEII* genes were upregulated in the transgenic lines (Fig. 4a). In addition, the mRNA levels of the genes encoding α-amylase and β-amylase, two starch-degrading enzymes, were significantly decreased (Fig. 4a).

The corresponding activities of AGPase, GBSSI, SSI, and SBEs increased significantly in transgenic lines by 1.14- to 1.26-, 2.03- to 2.57-, 1.69- to 1.96-, and 1.16- to 1.32-fold, respectively, compared with those in the WT (Fig. 4b), whereas α-amylase and β-amylase activities significantly decreased in the IA lines (Fig. 4b). Taken together, these findings suggest that increases in the activities of AGPase, GBSSI, SSI, and SBEs lead to increased starch deposition in the tuberous roots of IA lines.

### *IbVP1* overexpression affects both granular starch size and morphology in sweet potato storage roots

**Fig. 5:**
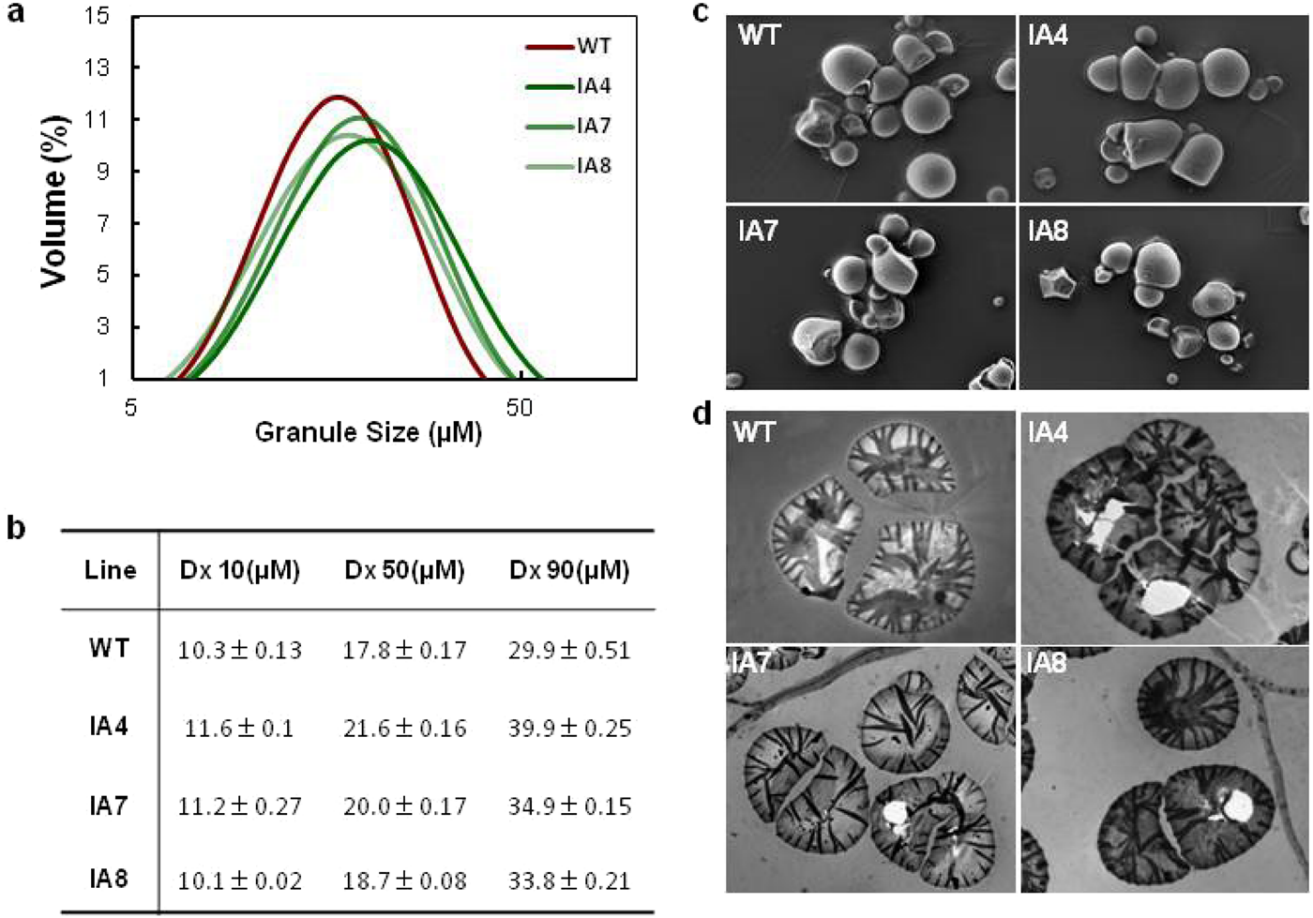
Starch granules from sweet potato storage roots. **a** Starch granule size distribution. **b** Descriptive diameters Dx10, Dx50, and Dx90 of starch granules. Dx10, Dx50, and Dx90 are the projected equivalent diameters below which 10%, 50, and 90% of the total volume of all particles analyzed is represented. **c** Scanning electron microscopy images of extracted starch. **d** Transmission electron microscopy images of starch granules in storage roots. WT wild type; IA4, IA7, and IA8 are three independent *IbVP1* transgenic lines. Five-month-old storage roots harvested from the field were used for analysis

The starch extracted from the storage roots of IA lines showed a broad distribution of granular starch size, ranging from 5.2 to 58.9 μm in the IA lines compared to 5.2–51.9 μm in the WT, and the average granule sizes of the IA lines were higher than those of the WT (Fig. 5a). Furthermore, the representative granule diameters, Dx10, Dx50, and Dx90, in the IA lines were 11, 20.1, and 36.2 μm, respectively, which were significantly higher than the 10.3, 17.8, and 29.9 μm diameters, respectively, of granules from the WT storage roots (Fig. 5b).

SEM analysis did not reveal any significant differences among the starch granule shapes between the IA lines and WT. Different granule shapes (oval, round, or polygonal) within the starch were observed for both IA lines and WT (Fig. 5c). However, some cracks were observed in the starch isolated from the IA roots, which is consistent with that observed previously in high-amylose sweet potato starch, as high amylose content disrupts the structure of the starch semicrystalline structure^26,37^. The cross-sections of sweet potato tuberous roots were examined under TEM, and WT starch granules revealed “zebra stripe” patterns, whereas those from IA transgenic lines showed darker black stripes (Fig. 5d).

### *IbVP1*-overexpressing sweet potato plants present altered starch composition and properties

**Fig. 6:**
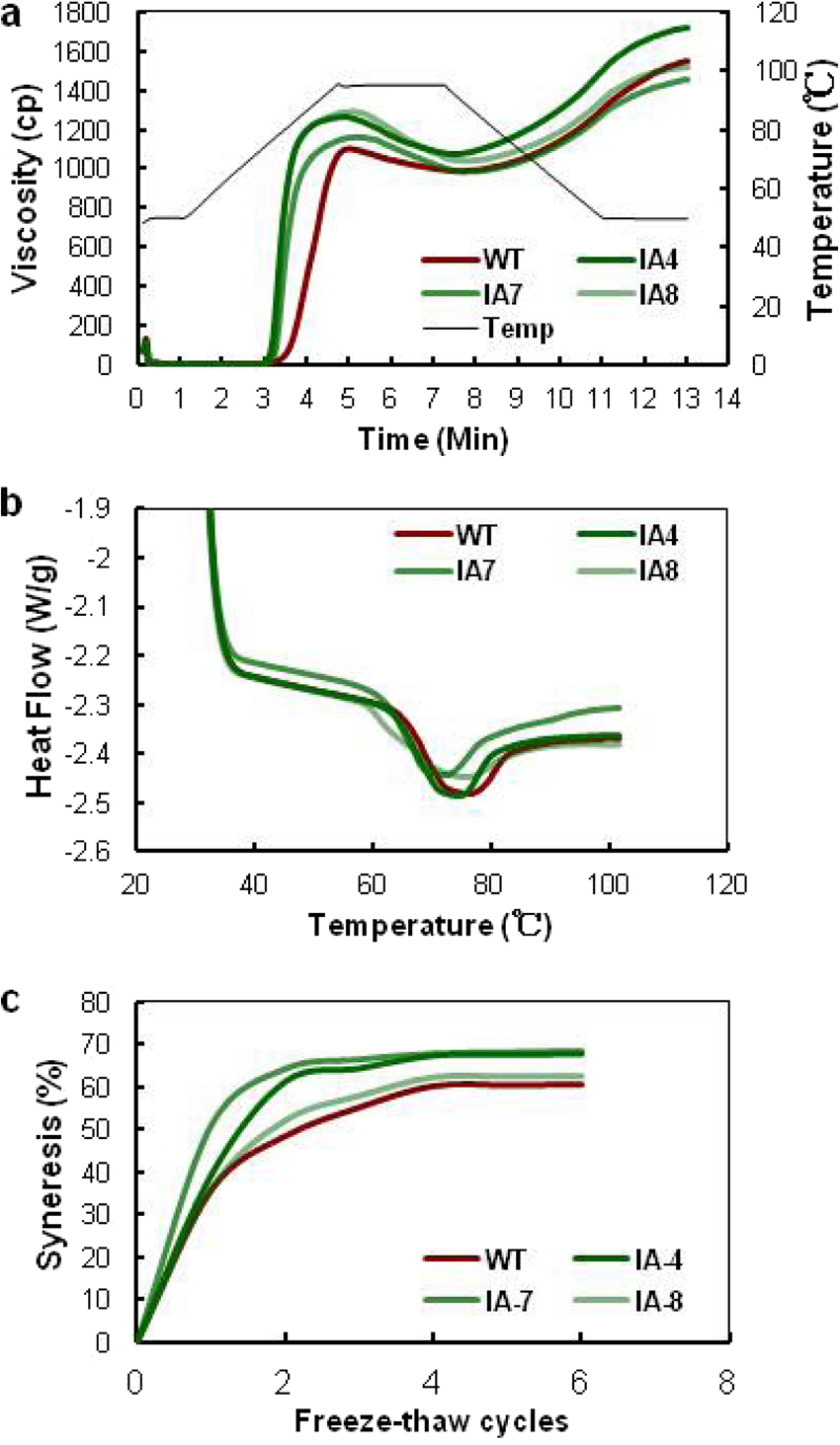
Physicochemical properties of sweet potato starch. **a** Pasting profiles of 5% starch suspension analyzed using a Rapid Visco-Analyzer. **b** Thermograms of starch analyzed using a differential scanning calorimeter. **c** Freeze–thaw stability assay of starch. WT wild type; IA4, IA7, and IA8 are independent *IbVP1* transgenic lines. Five-month-old storage roots harvested from the field were used for analysis

The pasting profiles of IA and WT starch were measured with RVA. As the temperature increased, granules of the WT starch swelled, with a rapid increase in viscosity; the granule structures of starch also became distorted, resulting in polymer dispersion, and finally, starch gel formed upon cooling. As shown in Fig. 6a, the characteristic curves of the IA starch were dramatically different from those of the WT starch, with the former exhibiting no discernible peak. We also measured the pasting property parameters, i.e., peak viscosity, final viscosity, breakdown, peak time, setback, and pasting temperature (Table S1). The viscosity peaks ranged from 1159 cP to 1290 cP for the IA starch; IA7 starch presented the lowest viscosity peak (1159 cP), which was still higher than that of the WT (1101 cP). The lowest breakdown values ranged from 175 cP (in IA7) to 252 cP (in IA8), and both were higher than the 114 cP of the WT (Table S1). The final viscosity was higher in the IA plants than the WT. However, the pasting temperatures of the IA starch were lower than those of the control starch.

Clear differences in onset temperature (*T*_o_), top melting temperature (*T*_p_), end melting temperature (*T*_e_), and enthalpy (Δ*H*) were observed in the IA starch compared with the WT starch. During the melting process, the *T*_o_ decreased slightly for all IA starch, ranging from 63.5 °C (in IA4) to 60.32 °C (in IA8), compared to the WT *T*_o_ of 64.77 °C, with a Δ*H* of 11.32 J g^−1^. Notable decreases in *T*_p_ and *T*_e_ were also detected in the IA starch compared to the WT starch (Fig. 6b). To determine the freeze–thaw stability in sweet potato starch, the level of syneresis of 5% starch paste was measured by repeating several cycles of freezing/thawing. Our results showed that, compared with the WT starch, the IA starch displayed significantly higher syneresis, even after just one cycle, ranging from 36.13–50.96%, with a maximum syneresis of 60.8% (Fig. 6c).

**Fig. 7:**
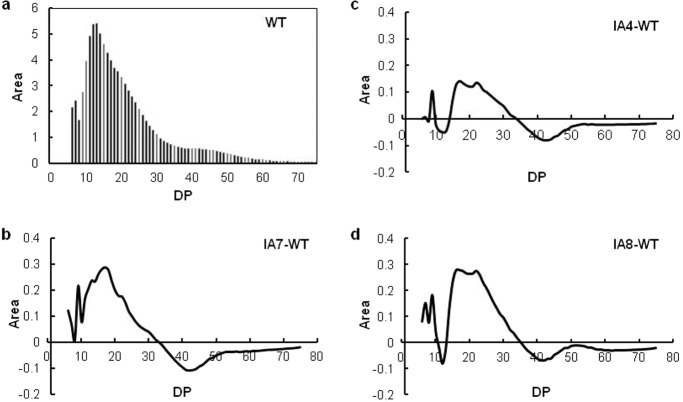
Amylopectin chain length distribution in sweet potato starch. **a** Degree of polymerization (DP) in WT starch. **b**–**d** Differences in chain length distribution between the IA lines and WT. IA4, IA7, and IA8 are independent *IbVP1*-overexpressing transgenic lines. The starch was extracted from storage roots of 5-month-old plants grown in the field

Amylopectin CLD was measured in isoamylase-treated starch samples using HPAEC-PAD analysis. The degree of polymerization (DP 6–75) was determined and normalized based upon the peak area (Fig. 7). The patterns of the amylopectin peaks were quite consistent among all the tested samples despite their different levels. Notably, the first trough was observed at DP 8, while two peaks were observed at DP 11–13 and at approximately DP 43, and there was a slight hump at DP 18; these results are consistent with previous findings^26,38,39^. To verify the DP differences, the amylopectin glucan chain value of WT was deducted from the corresponding IA starch (Fig. 7b–d). The chain lengths of DP 10–14 and DP 34–75 were reduced, whereas the DP 15–23 chains increased in the IA lines. Comparative starch analysis was performed, and it was noted that, compared with the WT starch, the IA starch comprised fewer short chains (maximum, −0.1%) and slightly more long chains (maximum, 0.28%).

## Discussion

Root crop productivity is largely determined by the ratio of acquisition and partitioning of organic carbon to sink organs. Previously, the roles of H^+^-PPases in many plants were reported to be associated with enhancing shoot and root growth by the efficient delivery of increased amounts of fixed carbon to sink tissue^18–25^. Although starch is the major form of fixed carbon in root crops, the roles of *IbVP1* in the storage roots of root crops have not yet been described. In our study, we found that overexpression of *IbVP1* in sweet potato resulted in increased photosynthesis ability of and sucrose contents in source leaves and promoted phloem loading and sucrose transport to sink organs, which in turn increased the total starch content in and yield of storage roots (Figs. 1–3). Gene expression and the activities of starch biosynthesis enzymes were concurrently upregulated (Fig. 4). *IbVP1*-induced *AGPase* gene expression is perhaps particularly critical for increased starch biosynthesis and total starch content because AGPase is the rate-limiting enzyme in starch metabolism^40,41^. *IbVP1* overexpression increased storage root numbers of the transgenic plants compared to the WT plants, possibly due to increased source-to-sink sucrose loading and transport for starch biosynthesis, triggering the development of some fibrous roots into storage roots^36^. Our results illustrate the important roles of *IbVP1* in increasing yield and increasing storage root numbers.

Sufficient carbohydrates from photosynthesis and high rates of phloem loading and sucrose transport are essential for the partitioning of photoassimilates into sink organs for plant production. Our data indicated that *IbVP1* enhances the net photosynthesis of leaves and influences sucrose translocation (Fig. 2) by assisting sucrose loading into phloem, thus resulting in more efficient sucrose transport from source to sink organs. In addition, upregulated expression of the sucrose proton symporter *SUT1* in sweet potato leads to higher sucrose levels in storage roots, suggesting that improved sucrose transport from the leaves to the roots enhances carbon fixation in storage roots (Figs. 1 and 3)^11^. Similarly, transgenic *Arabidopsis* lines with phloem companion cell-specific expression of *Arabidopsis* Vacuolar Pyrophosphatase (AVP) consistently have more hexose and starch in rosettes due to improved phloem transport accompanied by upregulated *Suc* gene expression^17^. Overexpression of the *AVP1* gene in *Arabidopsis* and barley results in an increase in phloem loading, transport, and unloading of sucrose into sink organs, which leads to biomass enhancement^19,22,42^. Improved transport of sugars from source to sink in H^+^-PPase-overexpressing plants may explain the increased biomass production as well as increased fixed carbon in the root system^21,25^. In our study, compared with the field-grown WT plants, the field-grown IA transgenic lines showed enhanced growth and vigor as well as increased yields of and total starch contents in the storage roots (Fig. 1). Sweet potato plants overexpressing *IbVP1* show enhanced levels of sucrose transport from source to sink, which likely affects auxin transport and increases rhizosphere acidification, leading to improved nutrient and water uptake^19^. Compared with the WT, the transgenic plants had 41 and 10% higher Pi concentrations in the leaves and roots, respectively, and the yields of storage roots of the IA lines were 20–39% higher than those of the WT in the field. In rice, overexpression of H^+^-PPase results in root systems that are more robust than those of the WT under both Pi-sufficient and Pi-deficient conditions^17^, which signifies the role of H^+^-PPase in optimizing Pi-use efficiency in plants^17,25^.

In addition to the increased total starch content of the IA lines, the ratio of amylose to amylopectin changed, which likely affects the physicochemical and functional properties of starch^43,44^. The amylose content peaked at 28.67% in the IA lines compared with 21.75% in the WT (Fig. 3c). In the IA lines, the expression of *GBSSI*, which is responsible for amylose biosynthesis, seems to be higher than the expression of *SBEI* and *SBEII*, which are responsible for amylopectin biosynthesis. This differential expression may explain the increase in the relative proportion of amylose. Furthermore, starch properties, including pasting properties and gelatinization, were altered in the IA lines (Figs. 5–6). We observed increased gelatinization temperature, lower breakdown, and higher setback values in starch from the IA lines compared with the WT. These values are similar to the high-amylose starch in the SBE-RNAi lines of sweet potato, which have higher proportions of long-chain amylopectin and larger granule sizes^26^. High amylose starch is desirable in some food applications because it is highly resistant to digestion and has a lower absorbance response^43,44^, thereby reducing health problems such as obesity and diabetes^45,46^.

**Fig. 8:**
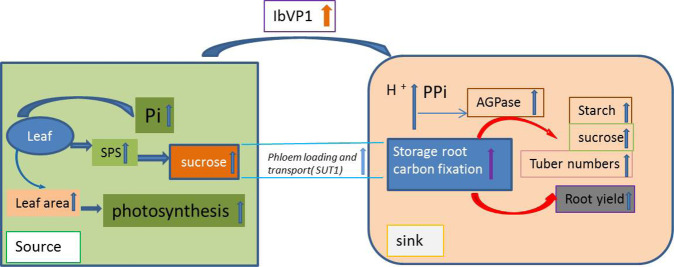
Model demonstrating the function of *IbVP1* in sweet potato. Activity of IbVP1 promotes inorganic Pi accumulation in cells, which increases the photosynthesis ability due to an enlarged leaf area and improved SPS activity and sucrose transport in the leaves. Increased sucrose levels are accompanied by enhanced phloem loading and transport, which result in increases in storage root carbon fixation, AGPase activity, and starch biosynthesis. SPS sucrose phosphate synthase, PPi inorganic pyrophosphate

In summary, sweet potato is an important root crop, and increasing yield and improving starch quality, especially increased storage root numbers and increased amylose contents, are difficult objectives to achieve through traditional breeding. Prior to this study, it was unclear how IbVP1 functions in the storage roots of sweet potato. Our study showed that IbVP1 plays a significant role in starch metabolism by altering the carbon metabolism flux to improve starch metabolism and increase yields of storage roots by influencing Pi metabolism. Enhanced phloem loading and long-distance transport of sucrose, mediated by IbVP1, increase yields and total starch content by mobilizing more photoassimilates from sources to sink organs (storage roots) for sink biomass production through increased photosynthesis, enlarged leaf areas, improved SPS activity, and decreased starch levels in the leaves of *IbVP1*-overexpressing transgenic lines. Overexpression of *IbVP1* increased rhizosphere acidification by increasing plasma membrane H^+^-ATPase activity to promote nutrient acquisition and auxin transport, contributing to improved plant growth, especially under stress conditions^11^. We suggest that IbVP1 functions to enhance sucrose transport mediated by the sucrose proton symporter SUT1 and to increase starch biosynthesis ability by altering pathway gene expression, thus altering carbon metabolism and starch physicochemical properties. These outcomes lead to enhanced carbohydrate transport from source organs to sink organs, increasing both root development and starch content (Fig. 8). The substantial improvement in yield due to increased tuber numbers and starch quality of the IA lines implies a practical approach to breeding sweet potato by manipulation of *IbVP1*.

## Supplementary information

supplemental file
