# Supplementary material for: The H+-pyrophosphatase IbVP1 regulates carbon flux to influence the starch metabolism and yield of sweet potato
Source: Hortic Res. 2021 Feb 1;8:20. doi: 10.1038/s41438-020-00454-2 (PMC7847997; doi:10.1038/s41438-020-00454-2)
Supplement: Supplementary file 1 — supplemental file [file 41438_2020_454_MOESM1_ESM.docx]

**Fig. S1** **Comparison of yield of wild-type and transgenic IbVP1 sweet potato plant (2014 in Wushe farm).**


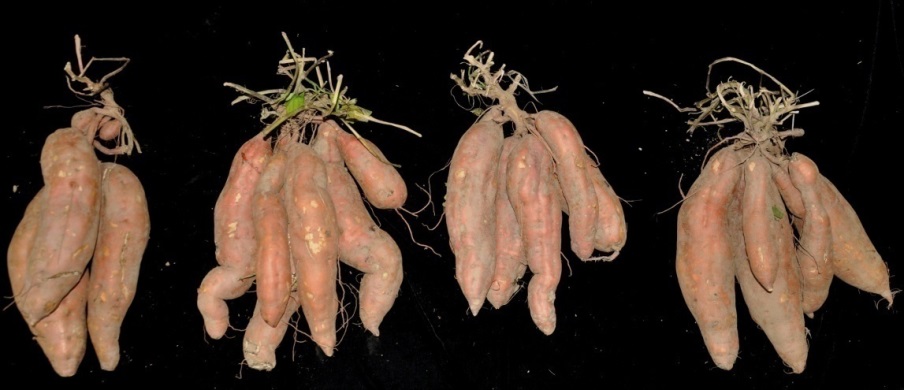
**a**

**WT IA4 IA7 IA8**

**b**

**
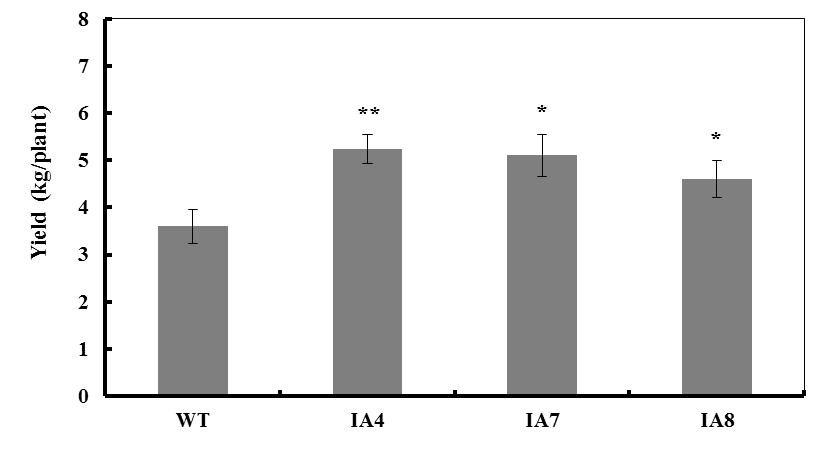
**

**c**

**Fig. S2 Sucrose and starch content were measured in wild-type and transgenic IbVP1 sweet potato plants**

**
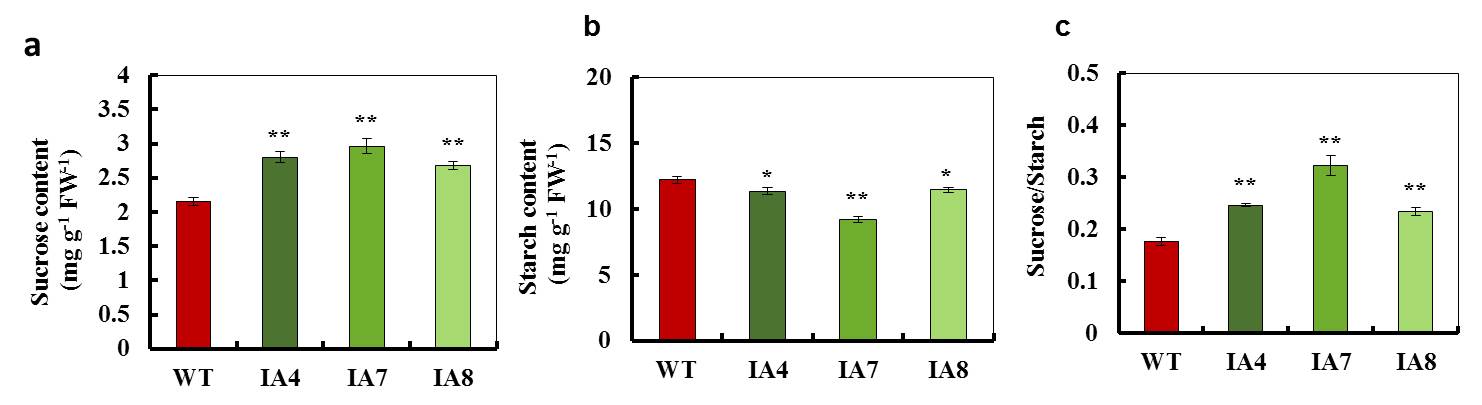
**

**Table S1**

**Rapid Visco-Analyser pasting profiles of starches from sweet potatoes**

| **Line** | **Peak 1** | **Breakdown** | **Final Viscosity** | **Setback** | **Peak Time** | **Pasting Temp** |
| --- | --- | --- | --- | --- | --- | --- |
| WT | 1101 | 114 | 1549 | 562 | 5 | 79.9 |
| IA4 | 1270 | 192 | 1725 | 647 | 4.87 | 75.2 |
| IA7 | 1159 | 175 | 1451 | 467 | 5.13 | 75.9 |
| IA8 | 1290 | 252 | 1516 | 478 | 5.07 | 75.95 |

**Table S2**

**Thermal characterization of starch of *IbVP1* overexpressed sweet potato**

|  | **To(℃)** | **Tp(℃)** | **Tc(℃)** | **△H(J/g)** |
| --- | --- | --- | --- | --- |
| WT | 64.77±1.18 | 72.51±0.13 | 82.29±0.07 | 11.32±0.57 |
| IA-1 | 63.5±0.81 | 71.32±0.16 | 80.14±0.30 | 10.57±0.66 |
| IA-4 | 61.74±0.08 | 68.63±0.15 | 78.15±0.37 | 10.91±1.06 |
| IA-8 | 60.32±1.78 | 70.72±1.23 | 79.32±2.40 | 9.93±0.80 |

T_o_： Onset temperature, T_p_： Peak temperature，T_c_： Conclusion temperature，∆H： Thermal enthalpy

**Table S3**

**qRT-PCR primers for genes related to starch metabolism pathways**

| **Primer** | **Forward primer (5’-3’)** | **Reverse primer (5’-3’)** |
| --- | --- | --- |
| *IbAGPa* | TCGACGGTGATGTTAGCAAG | AACAGCCTTTGGAGAAACGA |
| *Ibα-Amylase* | CTGCATTTTTGTTCCTGCAA | TTCGATGCGTCCAAGTCATA |
| *Ibβ-Amylase* | AGACTGGAAGGAGGCTGTGA | TGTTGGCTTCTTCGAGGACT |
| *IbGBSSI* | CAGTTGGTTTGCCAGTTGAC | ACGTTGAACTTTGCCACTCC |
| *IbSBEI* | GGTTTACGGGTCTTGATGGA | AACAGCCTGCTATCCCACAC |
| *IbSBEII* | CTTCCCTGAAGCCATAACCA | CCATTTGCCAATCCTCATCT |
| *IbSS* | CGGTTCACTTTGCTTTGTCA | CATTGTGTGGGCGATACTTG |
| *IbActin* | CTGGTGTTATGGTTGGGATGG | GGGGTGCCTCGGTAAGAAG |
